# Variation at *APOE *and *STH *loci and Alzheimer's disease

**DOI:** 10.1186/1744-9081-2-13

**Published:** 2006-04-07

**Authors:** Lingjun Zuo, Christopher H van Dyck, Xingguang Luo, Henry R Kranzler, Bao-zhu Yang, Joel Gelernter

**Affiliations:** 1 Division of Human Genetics, Department of Psychiatry, Yale University School of Medicine, New Haven, CT, USA; 2 VA Connecticut Healthcare System, West Haven Campus, CT, USA; 3 Alzheimer's Disease Research Unit and Cognitive Disorders Clinic, Department of Psychiatry, Yale University School of Medicine, New Haven, CT, USA; 4 Department of Psychiatry, University of Connecticut School of Medicine, Farmington, CT, USA

## Abstract

**Background:**

The apolipoprotein E (APOE) and tau proteins play important roles in the pathological development of Alzheimer's disease (AD). Many studies have shown an association between the *APOE *gene and AD. Association between AD and the newly discovered saitohin (*STH*) gene, nested within the intron of the tau gene, has been reported. The present study aimed to elucidate the association between *APOE *and AD, and between *STH *and AD in our sample.

**Methods:**

The functional polymorphisms, rs429358 and rs7412, in the *APOE *gene (which together define the *ε*2, *ε*3, and *ε*4 alleles), and the Q7R SNP in the *STH *gene, were genotyped in 369 patients with AD and 289 healthy European-Americans. The associations between these two genes and AD were analyzed in a case-control design.

**Results:**

Consistent with previously reported results, the frequencies of the *APOE ε*4 allele, *ε*4/*ε*4 genotype and *ε*3/*ε*4 genotype were significantly higher in AD cases than controls; the *ε*4/*ε*4 genotype frequency was significantly higher in early-onset AD (EOAD) than late-onset AD (LOAD); the frequencies of the *ε*2 allele, *ε*3 allele, *ε*3/*ε*3 genotype and *ε*2/*ε*3 genotype were significantly lower in AD cases than controls. Positive likelihood ratios (LRs^+^) of *APOE *alleles and genotypes increased in a linear trend with the number of *ε*4 alleles and decreased in a linear trend with the number of *ε*2 or *ε*3 alleles. There was no significant difference in the *STH *allele and genotype frequency distributions between AD cases and controls.

**Conclusion:**

This study confirmed that the *ε*4 allele is a dose-response risk factor for AD and the *ε*4/*ε*4 genotype was associated with a significantly earlier age of onset. Moreover, we found that the *ε*2 allele was a dose-response protective factor for AD and the *ε*3 allele exerted a weaker dose-response protective effect for risk of AD compared with *ε*2. In a clinical setting, *APOE *genotyping could offer additional biological evidence of whether a subject may develop AD, but it is not robust enough to serve as an independent screening or predictive test in the diagnosis of AD. *STH *variation was not significantly associated with AD in our sample.

## Background

Alzheimer's disease (AD) is the most common cause of dementia. It is a primary neurodegenerative cerebral disease in the elderly, characterized by two major histopathologic changes in the brain, i.e., extracellular amyloid plaques and intracellular neurofibrillary tangles [1,2].

**Figure 1:**
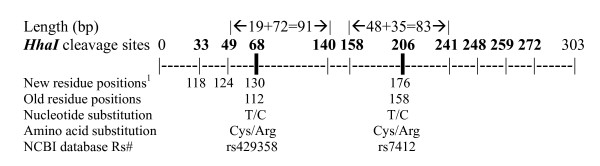
*HhaI *cleavage sites within the *APOE *amplicon. ^1 ^The residue positions are updated in the NCBI SNP database . *ε*2 allele (haplotype): 130Cys+176Cys (old:112Cys+158Cys) = 91bp+83 bp+others (≤ 33 bp); *ε*3 allele (haplotype): 130Cys+176Arg (old:112Cys+158Arg) = 91 bp+ 48 bp+35 bp+others (≤ 33 bp); *ε*4 allele (haplotype): 130Arg+176Arg (old:112Arg+ 158Arg) = 72+48+35+19+others (≤ 33 bp).

Apolipoprotein E (APOE) is one of the major cholesterol transport proteins. It exists in three major isoforms, APOE2, APOE3 and APOE4. The three APOE isoforms differ in the 112^th ^and 158^th ^residues of their primary structures (Figure 1); these differences are classified as SNPs rs429358 and rs7412, respectively. The APOE3 protein has higher receptor affinity than the variant types APOE2 and APOE4. Substitution of the basic amino acid Arg158 in APOE3 by the neutral amino acid Cys158 in APOE2 results in the receptor affinity of APOE2 being reduced to 2% of that of APOE3 [3]. In the central nervous system, APOE mediates the uptake and redistribution of cholesterol, and different APOE isoforms modify cholesterol homeostasis by preferentially associating with specific lipoprotein particles [4]. The role of APOE in modifying cholesterol homeostasis in the brain may contribute to the relationship between APOE and AD. Furthermore, APOE exists inside the amyloid plaque, where it can bind to *β*-amyloid (A*β*), which is a major component of the plaque [5]. Studies have shown that APOE interacts with A*β *to form a stable complex, altering the deposition of A*β *and affecting A*β*-induced neurotoxicity [6].

Moreover, APOE may be involved in Alzheimer's disease through a tau pathway. Studies have indicated that tau plays an important role in the physiopathology of Alzheimer's disease and that an extended haplotype (H1), covering the entire tau gene, including a 238 bp insertion in intron 9, is associated with AD [2,7-12], although these observations have not always been confirmed by other studies. APOE2 and APOE3 can bind to tau and prevent tau from being hyperphosphorylated. Although APOE4 also binds to tau, it cannot prevent tau from hyperphosphorylation, but destabilizes tau. The hyperphosphorylated tau can decrease tau's affinity for microtubules and severely disrupt microtubule stability, which has been postulated to be an important step in the formation of the paired helical filament (PHF) involved in neuronal degeneration. This may be part of the mechanism of APOE's important role in the etiology of AD.

APOE2, APOE3 and APOE4 are encoded by the *ε*2, *ε*3 and *ε*4 alleles, respectively [13]. The roles of these alleles in modulating risk for AD have been widely studied. (a) The *ε*4 allele contributes to the risk for AD across most populations [e.g., [14,15]]. (b) AD patients with the *ε*4 allele have an earlier age-of-onset than those without the *ε*4 allele [e.g., [16,17]]. (c) The *ε*4 allele has a significant dose effect on the risk for AD [e.g., [18-20]]. (d) The *ε*2 allele may protect individuals from being affected with AD [e.g., [14,15]].

Saitohin (*STH*), an intronless gene, has been shown to be nested in the intron between exons 9 and 10 of the tau gene, 2.5 kb downstream from exon 9. This region is functionally critical due to the splicing of exon 10. The special location of the *STH *gene has prompted investigation into its possible role in AD and other neurodegenerative disorders. The A224G polymorphism in the *STH *gene, which causes a glutamine (Q) to arginine (R) substitution at residue 7 (Q7R), is in linkage disequilibrium with the extended tau H1/H2 haplotype [21,22]. That is, the *STH *Q allele is associated with tau haplotype H1, and the *STH *R allele is associated with haplotype H2. An initial study by Conrad et al. [23] demonstrated that the Q7R polymorphism in the *STH *gene was associated with risk for AD. The *STH *gene has also been associated with autosomal dominant frontotemporal dementia (FTD), progressive supranuclear palsy (PSP) and Pick's disease [21,23-25]. Nevertheless, these findings remain controversial [22,25-29].

The purpose of the present study was to elucidate the associations between the variants at *APOE *and *STH *loci and AD in our samples, and to explore the gene-dose effects and evaluate the implications of variation at the *APOE *gene in the diagnosis of AD.

## Methods and materials

### Subjects

The sample included 658 European-Americans, including 369 patients with AD and 289 healthy controls. The diagnosis of AD was based on criteria of the National Institute of Neurological and Communicative Disorders and Stroke and Alzheimer's disease and Related Disorders Association (NINCDS-ADRDA) [30]. The AD cases were divided into an early-onset (EOAD) group and a late-onset (LOAD) group based on an age-of-onset of 70 years [27,29]. Each subject was evaluated for an approximate date of AD onset, based on careful review of medical records and detailed interviews with one or more primary caregivers. The date of onset was operationally defined as the date at which the "earliest definite AD symptom" appeared. The mean age of AD patients was 73.6± 8.4 years (range: 51.8 to 92.7); the mean age-of-onset was 69.3± 8.3 years (range: 44.6 to 86.7; 3 subjects unknown); 143 were male, 226 were female; 180 had positive family history (FH^+^), 184 had negative family history (FH^-^), and 5 had unknown family history. Family history of AD was assessed using the Alzheimer Dementia Risk Questionnaire (ADRQ) [31] and the Dementia Questionnaire (DQ) [32]. Family history was considered positive if at least one first-degree relative met criteria for primary degenerative dementia. No cases suggestive of autosomal dominant transmission were identified.

There were two sets of control subjects who were differentiated based on the method of ascertainment. The first set of healthy controls (n = 185) was recruited through advertisements in the community. They were screened using the Structured Clinical Interview for DSM-III-R (SCID), the Computerized Diagnostic Interview Schedule for DSM-III-R (C-DIS-R), the Schedule for Affective Disorders and Schizophrenia (SADS) [33], or an unstructured interview, to exclude major Axis I disorders, including substance dependence, psychotic disorders, mood disorders, anxiety disorders and dementia. Their mean age was 28.1± 9.1 years (range: 18.0 to 52.0); 81 were male and 104 were female. The second set of healthy controls (n = 104) was recruited primarily from among spouses of AD patients. Their mean age was 63.3± 16.3 years (range: 21.1 to 87.5); 49 were male and 55 were female. They were evaluated as being in generally good medical health for their age on the basis of a comprehensive evaluation that included medical history, physical and neurological examinations, serum chemistries, thyroid function studies, complete blood count, B12, folate, VDRL, urinalysis, electrocardiogram, and brain MRI or CT. The second set of controls was also required to have no significant evidence of cognitive impairment, as indicated by a Mini-Mental State Examination (MMSE) [34] score > 27. Subjects were recruited at Yale University School of Medicine, the University of Connecticut Health Center, or the VA Connecticut Healthcare System, West Haven Campus. Informed consent was obtained from all the patients and the controls. This study was performed after approval by the appropriate Institutional Review Boards (IRBs).

### Genotyping

Genomic DNA was extracted from peripheral blood by standard methods. The region flanking the two target markers within exon 5 of *APOE*, rs429358 and rs7412, was amplified by a single polymerase chain reaction (PCR) using the following primers [35]: APOE-A: 5'-CGGGCACGGCTGTCCAAGGAG-3' and APOE-C: 5'-CACGCGGCCCTGTTCCACgAG-3' (g is mismatched). PCR was performed in a final volume of 10 μl with 1× PC2 buffer (Ab Peptides, Inc., St. Louis, MO), 1M betaine, 0.5 units of KlenTaq polymerase (Ab Peptides, Inc., St. Louis, MO) and 10 ng DNA. PCR conditions were set as follows: 95°C for 5 min; 35 cycles of 95°C for 30 s, 64°C for 30 s, and 72°C for 30 s. The genotypes were analyzed on 5% metaphor agarose gel after digestion with *HhaI *(New England Biolabs Inc., Beverly, MA). The size of the PCR product was 303 bp, within which there are eight constant *HhaI *cleavage sites (GCG|C) and two variant *HhaI *cleavage sites (see Figure 1).

The region flanking the Q7R marker in the *STH *gene was amplified by PCR using the primers from the initial study by Conrad et al. [23]. PCR was performed in a final volume of 15 μl with 1× PC2 buffer, 1M betaine, 0.75 units of KlenTaq polymerase and 25 ng DNA. PCR conditions were set as follows: 95°C for 5 min; 30 cycles of 95°C for 30 s, 60°C for 30 s, and 72°C for 30 s. The genotypes were analyzed on 3% metaphor agarose gel after digestion with *HinfI *(New England Biolabs Inc., Beverly, MA). The size of the PCR product was 226 bp. The R allele (Arginine, CGA) can be cut by *HinfI *(97 bp+74 bp+55 bp), whereas the Q allele (Glutamine, CAA) cannot (171 bp+55 bp).

### Statistical analysis

The comparisons in allele and genotype frequency distributions between two groups were performed with Fisher's exact test. Bonferroni correction was used to adjust the *α *level of multiple comparisons [36].

Positive predictive values (PPVs) were calculated with Bayes' rule [37]. *P(AD) *was the prior probability of developing AD, i.e., the prevalence of AD (see Formula). We used 15% as the estimated prevalence of AD [38]; *P*(*Controls*) ≈ 1-*P*(*AD*); *P*(*ε*|*AD*) was allele or genotype frequency in AD cases, and *P*(*ε*|*Controls*) was allele or genotype frequency in controls. Both *P*(*ε*|*AD*) and *P*(*ε*|*Controls*) were estimated from the present study;  (*AD*|*ε*) was the posterior probability of developing AD given a certain allele or genotype.



Positive likelihood ratios (LRs^+^) were calculated by dividing the allele or genotype frequencies in AD cases by those in controls [39]. For example, if the frequency of the *ε*4/*ε*4 genotype is 0.139 in AD cases and 0.037 in controls, then the LR^+ ^is equal to 0.139/0.037 = 3.757.

The dose effect of the *APOE *gene, i.e., the relationship between the risk for AD and the number of *APOE *alleles, was tested by the chi-square test for trend using the software EPISTAT [40]. The relationships between the number of *APOE *alleles and their LRs^+ ^were tested with Spearman's rank correlation analysis implemented in SPSS 13.0 (SPSS Inc., Chicago, IL). Gene dose effects for *APOE *were plotted using a polynomial curve-fitting plot method in S-PLUS 2000 (Mathsoft Engineering & Education, Inc., Cambridge, MA).

Age, sex, and AD family history are confounders that may cause false positive or false negative results. Thus, we used stepwise logistic regression analysis to investigate the association between the risk for AD and the number of *APOE *and *STH *alleles, controlling for the effects of the potential confounders. In the stepwise logistic regression model, the diagnosis served as the dependent variable; the independent variables included the number of *APOE ε*4 alleles, the number of *APOE ε*2 alleles, the number of *STH *R alleles, the interaction between *STH *R allele and *APOE *alleles, age, sex and AD family history. This analysis was performed with SPSS 13.0 software.

## Results

There was no significant difference in allele frequency distributions, genotype frequency distributions or dose effects of *APOE *and *STH *gene between our two sets of controls, so we combined the two control groups into one larger control group.

### Associations of *APOE *alleles and genotypes with Alzheimer's disease

The comparisons of allele and genotype frequency distributions between AD cases and controls are shown in Tables 1 and 2. The genotype frequency distributions in both AD cases and controls were in Hardy-Weinberg equilibrium (HWE).

**Table 1:** Distributions of allele frequencies of *APOE *and *STH *gene variations in European-American subjects

	*APOE *alleles							Exact p-values				*STH alleles*					Exact p-values	
					
	*ε*2		*ε*3		*ε*4		Total	*ε*2	*ε*3	*ε*4	Total	Q		R		Total		
													
	*f*	n	*f*	n	*f*	n	2N					*f*	n	*f*	n	2N		
**AD**	0.024	17	0.617	443	0.359	258	718	0.001	6.0*10^-12^	2.4*10^-18^	1.2*10^-18^	0.767	439	0.233	133	572	0.345	1
EOAD	0.017	6	0.590	203	0.392	135	344	0.002	4.7*10^-11^	6.8*10^-17^	2.5*10^-17^	0.771	216	0.229	64	280	0.506	2
LOAD	0.027	10	0.644	237	0.329	121	368	0.022	3.6*10^-7^	3.1*10^-11^	5.5*10^-11^	0.762	218	0.238	68	286	0.348	2
FH^+^	0.014	5	0.590	204	0.396	137	346	6.8*10^-4^	4.9*10^-11^	2.1*10^-16^	2.3*10^-17^	0.737	205	0.263	73	278	0.094	2
FH^-^	0.030	11	0.641	232	0.329	119	362	0.051	2.2*10^-7^	4.1*10^-11^	1.1*10^-10^	0.796	226	0.204	58	284	1.000	2
Male	0.032	9	0.635	179	0.333	94	282	0.093	3.9*10^-5^	1.7*10^-7^	3.9*10^-7^	0.738	155	0.262	55	210	0.145	3
Female	0.018	8	0.606	264	0.376	164	436	0.008	7.0*10^-8^	9.8*10^-12^	4.9*10^-12^	0.785	284	0.215	78	362	1.000	3
**Controls**	0.061	30	0.801	394	0.138	68	492					0.794	313	0.206	81	394		
Male	0.065	15	0.800	184	0.135	31	230					0.805	140	0.195	34	174		
Female	0.057	15	0.802	210	0.141	37	262					0.786	173	0.214	47	220		

*f, frequency; n, number of alleles; N, number of individuals; 2N, number of chromosomes; AD, Alzheimer's disease; EOAD, early-onset AD (≤70yrs); LOAD, late-onset AD (>70yrs); Controls, the total controls combining two subgroups through two different diagnostic evaluations. The numbers in last column denote: 1. Comparing overall AD with controls (α was set at 0.017 for each APOE allele comparison); 2. Comparing EOAD, LOAD, FH*^+^*AD and FH*^-^*AD with controls respectively (α was set at 0.003 for each APOE allele comparison); 3. Comparing male AD with male controls and female AD with female controls (α was set at 0.003 for each APOE allele comparison)*.

**Table 2:** Distributions of the genotype frequencies of the *APOE *gene variations in European-American subjects

	*APOE *genotypes											Exact P-values					
		
	*ε*2/*ε*3		*ε*2/*ε*4		*ε*3/*ε*3		*ε*3/*ε*4		*ε*4/*ε*4		Total	*ε*2/*ε*3	*ε*2/*ε*4	*ε*3/*ε*3	*ε*3/*ε*4	*ε*4/*ε*4	Total
												
	*f*	N	*f*	N	*f*	N	*f*	N	*f*	N	N						
**AD**	0.036	13	0.011	4	0.384	138	0.429	154	0.139	50	359	0.001	0.721	8.1*10^-11^	3.0*10^-10^	1.9*10^-5^	2.1*10^-16^
EOAD	0.029	5	0.006	1	0.390	67	0.372	64	0.203	35	172	0.004	0.653	1.1*10^-7^	2.9*10^-5^	5.8*10^-8^	2.5*10^-13^
LOAD	0.038	7	0.016	3	0.386	71	0.478	88	0.082	15	184	0.010	1.000	3.8*10^-8^	1.6*10^-10^	0.056	2.5*10^-11^
FH^+^	0.029	5	0.000	0	0.341	59	0.468	81	0.162	28	173	0.004	0.146	2.8*10^-10^	9.8*10^-10^	1.4*10^-5^	4.1*10^-16^
FH^-^	0.039	7	0.022	4	0.425	77	0.392	71	0.122	22	181	0.010	0.727	3.3*10^-6^	3.5*10^-6^	0.001	6.1*10^-9^
Male	0.043	6	0.021	3	0.390	55	0.447	63	0.099	14	141	0.053	1.000	1.8*10^-5^	1.8*10^-6^	0.099	6.3*10^-7^
Female	0.032	7	0.005	1	0.381	83	0.417	91	0.165	36	218	0.015	0.559	1.7*10^-6^	6.4*10^-5^	8.7*10^-5^	8.9*10^-10^
**EOAD vs. LOAD**												0.772	0.624	1.000	0.053	0.001	0.009
**Controls**	0.106	26	0.016	4	0.654	161	0.187	46	0.037	9	246						
Male	0.113	13	0.017	2	0.661	76	0.165	19	0.043	5	115						
Female	0.099	13	0.015	2	0.649	85	0.206	27	0.031	4	131						

*f, N, AD, EOAD, LOAD, Controls and the comparing methods see Table 1. EOAD vs. LOAD, comparing EOAD and LOAD. α was set at 0.010 (or 0.002) for each genotype comparison between overall AD and controls (or between AD subgroups and controls), referring to Table 1. ε2/ε3, the genotype consists of allele ε2 and allele ε3; as analogy*.

The overall allele and genotype frequency distributions in AD cases were significantly different from those in controls. The frequencies of the *ε*4 allele, *ε*3/*ε*4 and *ε*4/*ε*4 genotypes were significantly higher in AD cases than in controls and the frequencies of the *ε*2, *ε*3 alleles, *ε*2/*ε*3 and *ε*3/*ε*3 genotypes were significantly lower in AD cases than in controls.

We also compared allele and genotype frequencies in AD subgroups (EOAD, LOAD, FH^+ ^AD, FH^- ^AD, male AD and female AD) with those in controls. The overall allele and genotype frequency distribution in each of the AD subgroups was significantly different from that in controls. Specifically, the frequencies of the *ε*4 allele and the *ε*3/*ε*4 genotype in each of the AD subgroups, and the *ε*4/*ε*4 genotype in EOAD, FH^+ ^AD, FH^- ^AD, and female AD were significantly higher than those in controls; the frequencies of the *ε*3 allele and the *ε*3/*ε*3 genotype in each of the AD subgroups, and the *ε*2 allele in EOAD, FH^+ ^AD and female AD were significantly lower than those in controls. The genotype frequency distributions were significantly different between EOAD and LOAD [(the *ε*4/*ε*4 genotype frequency in EOAD (0.203) was significantly higher than that in LOAD (= 0.082)]. Among these differences, the nominal difference in the frequency of the *ε*2 allele between cases and controls was not statistically significant after Bonferroni correction.

Stepwise logistic regression analyses showed that after adjusting for age, sex, and AD family history, the *ε*4 and *ε*2 alleles were still significantly associated with risk for AD (*P*_*ε*4 _= 0.014, adjusted OR_*ε*4 _= 1.86,95% Cl_*ε*4_: 1.13–3.05; *P*_*ε*2 _= 0.041, adjusted OR_*ε*2 _= 0.36,95% Cl_*ε*2_:0.13–0.96).

### PPVs and LRs+ of the *APOE *gene for the diagnosis of AD

PPVs and LRs^+ ^of *APOE *alleles and genotypes for AD are listed in Table 3. Both PPVs and LRs^+ ^of *APOE *alleles and genotypes were in the following order: *ε*4/*ε*4 > *ε*4 > *ε*3/*ε*4 > *ε*3 > *ε*2/*ε*4 > *ε*3/*ε*3 > *ε*2 > *ε*2/*ε*3.

**Table 3:** Interpretation of PPVs and LRs^+ ^of *APOE *alleles and genotypes

allele/genotype	PPV [ (*AD*|*ε*)]	LR^+^	LR range^1^	Significance for diagnosis
			> 10	greatly increasing risk for AD
			5~ 10	moderately increasing risk for AD
*ε*4/*ε*4	39.90%	3.76	2~ 5	small increase in risk for AD
*ε*4	31.50%	2.60		
*ε*3/*ε*4	28.80%	2.29		
			1~ 2	minimally increasing risk for AD
	15.00% (population prevalence)		1	no change in risk for AD
*ε*3	12.00%	0.77	0.5~ 1	minimally decreasing risk for AD
*ε*2/*ε*4	10.82%	0.69		
*ε*3/*ε*3	9.40%	0.59		
*ε*2	6.50%	0.39	0.2~ 0.5	small decrease in risk for AD
*ε*2/*ε*3	5.70%	0.34		
			0.1~ 0.2	moderately decreasing risk for AD
			< 0.1	greatly decreasing risk for AD

*PPV, estimated positive predictive value; LR*^+^*, positive likelihood ratios*.

^1^*Reference to [39]*.

We also compared PPVs for different subtypes of AD. PPVs of the *ε*4/*ε*4 genotype were much higher in EOAD (49.2% vs. 28.1% for LOAD), female AD (48.4% vs. 28.9% for male AD) and FH^+ ^AD (43.6% vs. 36.8% for FH^- ^AD). In addition, the PPV for the *ε*3/*ε*4 genotype was higher in LOAD (31.1%) than EOAD (26.0%). Finally, the PPVs were lower for FH^+ ^AD for the *ε*2 allele and the *ε*2/*ε*3 genotype (3.9%, 4.6%, respectively) than for FH^- ^AD (8.0%, 6.1%, respectively).

### Gene dose effects of the *APOE *gene on the risk for AD (Table 4 and Figure 2)

**Table 4:** Distributions of the frequencies of the genotypes with ascending *APOE *allele number

	Number of *ε*2 alleles						Chi-Square test for trend		Number of *ε*3 alleles						Chi-Square test for trend		Number of *ε*4 alleles						Chi-Square test for trend	
						
	0		1		2		*χ*^2^	P	0		1		2		*χ*^2^	P	0		1		2		*χ*^2^	P
															
	*f*	N	*f*	N	*f*	N			*f*	N	*f*	N	*f*	N			*f*	N	*f*	N	*f*	N		
**AD**	0.953	342	0.047	17	0	0	11.32	0.001	0.150	54	0.465	167	0.384	138	43.02	< 0.0001	0.421	151	0.440	158	0.139	50		< 0.0001
EOAD	0.965	166	0.035	6	0	0	9.73	0.002	0.209	36	0.401	69	0.390	67	37.41	< 0.0001	0.419	72	0.378	65	0.203	35	56.72	< 0.0001
LOAD	0.946	174	0.054	10	0	0	5.69	0.017	0.098	18	0.516	95	0.386	71	26.27	< 0.0001	0.424	78	0.494	91	0.082	15	42.63	< 0.0001
FH^+^	0.971	168	0.029	5	0	0	11.46	0.001	0.162	28	0.497	86	0.341	59	41.06	< 0.0001	0.370	64	0.468	81	0.162	28	61.13	< 0.0001
FH^-^	0.939	170	0.061	11	0	0	4.49	0.034	0.144	26	0.431	78	0.425	77	24.67	< 0.0001	0.464	84	0.414	75	0.122	22	38.60	< 0.0001
Male	0.936	132	0.064	9	0	0	3.29	0.069	0.121	17	0.489	69	0.390	55	16.00	< 0.0001	0.433	61	0.468	66	0.099	14	24.98	< 0.0001
Female	0.963	210	0.037	8	0	0	8.02	0.005	0.170	37	0.450	98	0.381	83	26.33	< 0.0001	0.413	90	0.422	92	0.165	36	38.04	< 0.0001
**Controls**	0.878	216	0.122	30	0	0			0.053	13	0.293	72	0.654	161			0.760	187	0.203	50	0.037	9		
Male	0.870	100	0.130	15	0	0			0.061	7	0.278	32	0.661	76			0.774	89	0.183	21	0.043	5		
Female	0.885	116	0.115	15	0	0			0.046	6	0.305	40	0.649	85			0.748	98	0.221	29	0.031	4		

f, N, AD, EOAD, LOAD, Controls, and the comparing methods see Tables 1 and 2.

*α *was set at 0.017 (or 0.003) for each Chi-Square test for trend analysis in overall AD (or AD subgroups), referring to Tables 1 and 2.

**Figure 2:**
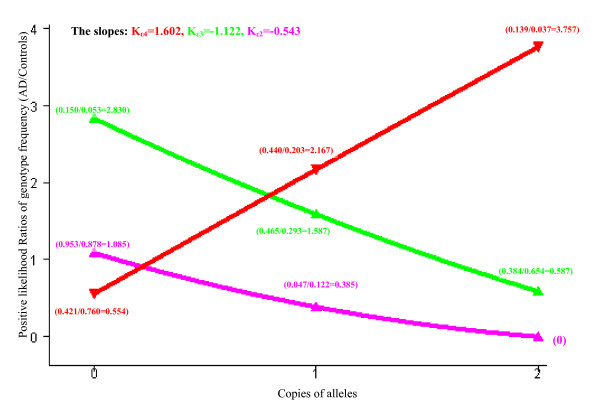
Dose effects of *APOE *gene alleles. X-axis represents the copies of alleles (*ε*2 allele, pink line; *ε*3 allele, green line; *ε*4 allele, red line); Y-axis represents the positive likelihood ratios of genotype frequency (in AD cases vs. in Controls)

The chi-square test for trend analyses showed that there was a significant positive correlation between the number of *ε*4 alleles and risk for AD and a significant negative correlation between the number of *ε*2 or *ε*3 alleles and risk for AD.

Similarly, the Spearman's rank correlation analysis showed that the number of *APOE *alleles was significantly correlated with LR^+^, which increased linearly with the number of the *ε*4 alleles (correlation coefficient r_*ε*4 _= 1.0; slope K _*ε*4 _= 1.602) and decreased linearly with the number of *ε*2 or *ε*3 alleles (correlation coefficient r_*ε*2 or *ε*3 _= 1.0; slope K_*ε*2 _= -0.543; slope K_*ε*3 _= -1.122).

### Association of the *STH *gene with AD

No significant difference in *STH *allele and genotype frequency distributions was found between AD cases and controls. Even after adjusting for potential confounding by the *APOE *gene, age, sex and AD family history, stepwise logistic analyses showed no association of *STH *alleles or genotypes with AD.

### Interactive effects between the *STH *gene and the *APOE *gene

Using *STH *genotypes, we grouped all subjects into QQ, RR and QR groups. We then compared *APOE *allele and genotype frequency distributions in these three groups in both cases and controls. No significant difference was found for any of the comparisons (data not shown).

## Discussion

The present study confirmed the well-established association between the *APOE *gene and AD. All three *APOE *alleles (*ε*2, *ε*3 and *ε*4) showed dose effects on the risk for AD, and followed a co-dominant mode of inheritance. We also examined, for the first time to our knowledge for a trait in neuropsychiatry, a mathematical measure of the predictive value of each *APOE *allele and genotype for AD diagnosis risk.

In addition to a significant association between the *APOE *gene and Alzheimer's disease, subgroup analyses revealed an association with subtypes based on age of onset, family history, and sex. The *ε*4 allele, the *ε*4/*ε*4 genotype and the *ε*3/*ε*4 genotype were risk factors for AD; the *ε*2 allele, the *ε*3 allele, the *ε*2/*ε*3 genotype and the *ε*3/*ε*3 genotype were protective factors for AD. These findings are consistent with those in most previous studies [e.g., [14,15]]. Further comparisons among AD subgroups and controls showed that the *ε*4/*ε*4 genotype frequency was significantly higher in EOAD than in LOAD and controls, suggesting that the *ε*4/*ε*4 genotype can significantly reduce the age-of-onset. This is consistent with findings in other studies [e.g., [19]].

We also found that the PPV of the *ε*4/*ε*4 genotype was significantly higher in females (48.4%) than in males (28.9%). Although the *ε*4/*ε*4 genotype frequency in female AD cases was significantly higher than in female controls, we found no significant difference in males. These results suggest that the *ε*4/*ε*4 genotype is a stronger risk factor for females than for males. This is consistent with findings from other studies [e.g., [41-43]]. However since sex distributions were not well matched between cases and controls, it could also reflect a stratification effect by sex.

Both the chi-square test for trend and the regression analyses revealed that the risk for AD increased significantly with the number of *ε*4 alleles. This is also consistent with findings from other studies [e.g., [18]]. In addition, we found that the risk for AD decreased with the number of *ε*2 or *ε*3 alleles. Furthermore, the dose of *APOE *alleles was linearly related to LR^+^. These results are all compatible with those from our allelewise analyses.

This information is of importance in predicting the development of AD in early life. However, not all subjects with the *ε*4 allele develop AD, nor do all AD patients carry the *ε*4 allele. On the other hand, not all subjects are protected against AD by the *ε*2 and *ε*3 alleles. Therefore, it is important to estimate the probability that these allele carriers will develop AD. We found that the *ε*4/*ε*4 genotype had a PPV of 39.90% and an LR^+ ^of 3.76 for AD. In other words, a subject carrying two *ε*4 alleles has a probability of 39.90% to develop AD. In contrast, a subject carrying one *ε*4 allele and one *ε*3 allele has a probability of 28.80% to develop AD, and a subject carrying one *ε*4 allele and one *ε*2 allele has a probability of 10.82% to develop AD. Based on the interpretation of LRs^+ ^by Ebell [39], the presence of *APOE *alleles can only mildly change the risk for AD, despite a highly significant association with AD. This implies that *APOE *genotype testing can provide evidence on whether a subject may develop AD, but it is not sufficient as an independent screening or predictive test for the diagnosis of AD [44]. Additionally, we found the following order for both PPVs and LRs^+ ^of *APOE *alleles and genotypes with respect to the diagnosis of AD: *ε*4/*ε*4 > *ε*4 > *ε*3/*ε*4 > *ε*3 > *ε*2/*ε*4 > *ε*3/*ε*3 > *ε*2 > *ε*2/*ε*3 (see Table 3). This order shows that: (1) *ε*4/*ε*4 > *ε*4, suggesting that the risk for AD increases with the number of *ε*4 alleles; (2) *ε*4 > *ε*3/*ε*4 and *ε*3/*ε*4 > AD population prevalence, suggesting that the *ε*3 allele reduces the risk for AD conveyed by the *ε*4 allele, but the protective effect of *ε*3 is weaker than the risk effect of *ε*4; (3) *ε*3/*ε*3 <*ε*3, suggesting that the protection against AD increases with the number of *ε*3 alleles; (4) *ε*3 > *ε*2/*ε*3 and *ε*2 > *ε*2/*ε*3, suggesting that the protective effect on AD risk for a genotype containing two protective alleles is greater than that for a genotype containing only one of the protective alleles; (5) *ε*3 > *ε*2 and *ε*3/*ε*3 > *ε*2/*ε*3, suggesting that the *ε*2 allele is a stronger protective factor for AD than the *ε*3 allele, which is reflected in their positions on the Y axis in the figure depicting the dose effect (Figure 2); and (6) *ε*3/*ε*4 > AD population prevalence, but *ε*2/*ε*3 <*ε*3/*ε*3 <*ε*3 < AD population prevalence, suggesting that without *ε*4, the *ε*3 allele and any genotypes containing the *ε*3 allele cannot increase risk for AD, that is, it is *ε*4, not *ε*3, that contributes to the increased risk of AD associated with the *ε*3/*ε*4 genotype. Similarly, the PPV for *ε*2/*ε*4 < AD population prevalence (i.e., a protective effect), but *ε*4/*ε*4 > *ε*4 > *ε*3/*ε*4 > AD population prevalence (i.e., a risk effect), suggesting that without *ε*2, the *ε*4 allele and any genotypes containing the *ε*4 allele (e.g., *ε*4/*ε*4 and *ε*3/*ε*4) do not have a protective effect; it is *ε*2, not *ε*4, that results in the *ε*2/*ε*4 genotype having a lower PPV. Taken together, the order of these effects suggests that *ε*4 is a dose-response risk factor for developing AD, *ε*2 is a dose-response protective factor, and *ε*3 is a relatively weaker dose-response protective factor. These findings are consistent with the results of our allelewise analyses, chi-square tests for trends, and logistic regression analyses.

There has been debate about whether the presence of a "bad" allele (i.e., *ε*4) or of a "good" allele (*ε*2 or *ε*3), or both, contribute to the association between *APOE *and AD. The answer to this question is important for the development of specific therapies for AD [45]. Our results tend to show that both the "bad" allele (*ε*4) and the "good" alleles (*ε*2 and *ε*3) are involved in the risk for AD, consistent with codominant inheritance. These findings are supported by the evidence from studies on the neuropathological processes involved in AD [e.g., [6]].

Noting both the close interaction between the APOE and the tau proteins and the physical proximity of the Tau and *STH *genes, we investigated the correlation between effects of the *APOE *and *STH *gene polymorphisms. We found no significant interactive effect between these two genes either in cases or in controls. This finding was consistent with our regression analysis and the studies by Conrad et al. [23] and Peplonska et al. [22]. Thus, the *APOE *gene affects risk for AD through a pathway independent of the *STH *gene polymorphism we queried.

We also found no associations between *STH *alleles and AD, even after adjusting for potential confounders, including age, sex, and family history. Neither the genotype analysis nor the gene-dose analysis showed any association. Our results suggest that *STH *may not be a risk gene for AD. The initial positive findings by Conrad et al. [23] may be attributable to sampling bias in the context of small sample sizes (51 AD cases; 30 healthy controls). Our sample size (286 AD cases; 197 healthy controls) is much larger than theirs. Moreover, our negative findings are in good agreement with many other studies, which also have much larger sample sizes (e.g., 499 AD cases and 402 controls by Verpillat et al. [25]; 225 AD cases and 144 controls by Streffer et al. [27]; 200 AD cases and 458 controls by Clark et al. [28]; 690 AD families, 903 AD cases and 320 controls by Oliveira et al. [29]; 100 AD cases and 100 controls by Peplonska et al. [22]). Additionally, the Q allele frequency (0.867) in controls in the initial study is similar to both controls and cases in our and the other negative studies; but the Q allele frequency (0.676) in AD cases is significantly lower than those in cases and controls in most of the published studies [22,25,27-29]. So far, there has been only one study [24] reporting a replicated positive finding between the genotype *STH *RR and AD (p = 0.04), but even this positive finding is only nominal and does not survive after Bonferroni correction. Therefore, we conclude that the *STH *gene Q7R variation does not play an important role in the pathology of AD.

## Competing interests

The author(s) declare that they have no competing interests.

## Authors' contributions

LZ designed the study, genotyped most of the subjects, analyzed and interpreted the data, and drafted the manuscript. CHVD collected the samples, participated in the design of the study, interpreted the data, and critically revised the manuscript. XL participated in designing the study, genotyping some subjects, analyzing and interpreting the data, and drafting the manuscript. HRK collected the samples, contributed to the interpretation, and made critical comments on the manuscript. BZY contributed to statistical analysis and data interpretation. JG obtained the funding, prepared the samples, supervised the study, interpreted the data, and made critical comments on the manuscript. All authors read and approved the final manuscript.
